# Anterior eye tissue morphology: Scleral and conjunctival thickness in children and young adults

**DOI:** 10.1038/srep33796

**Published:** 2016-09-20

**Authors:** Scott A. Read, David Alonso-Caneiro, Stephen J. Vincent, Alexander Bremner, Annabel Fothergill, Brittney Ismail, Rebecca McGraw, Charlotte J. Quirk, Elspeth Wrigley

**Affiliations:** 1 Contact Lens and Visual Optics Laboratory, School of Optometry and Vision Science, Queensland University of Technology, Brisbane, Queensland, Australia

## Abstract

The sclera and conjunctiva form part of the eye’s tough, protective outer coat, and play important roles in the eye’s mechanical protection and immune defence, as well as in determining the size and shape of the eye globe. Advances in ocular imaging technology now allow these tissues in the anterior eye to be imaged non-invasively and with high resolution, however there is a paucity of data examining the dimensions of these tissues in paediatric populations. In this study, we have used optical coherence tomography (OCT) imaging to examine the normal *in vivo* thickness profile of the anterior sclera and overlying conjunctiva in 111 healthy young participants, including a large proportion of paediatric subjects. We demonstrate that the thickness of the anterior sclera varies significantly with measurement location and meridian. Tissue thickness also varied significantly with age, with younger subjects exhibiting significantly thinner scleras and significantly greater conjunctival thickness. Males were also found to exhibit significantly greater scleral thickness. Refractive error however was not significantly associated with either scleral or conjunctival thickness in this population. These findings provide new data describing the normative dimensions of anterior eye tissues in children and the factors that can influence these dimensions in young populations.

The sclera is the eye’s robust, outer coat of connective tissue, composed primarily of collagen fibrils embedded in a hydrated matrix of proteoglycans^1^. It plays important biomechanical roles in protecting and stabilising the eye’s contents from both internal and external stresses and strains, and is one of the primary determinants of the size and shape of the eye globe^2^,^3^. Previous research, primarily involving animal models and *ex vivo* studies of human eyes, has documented changes in the structure and biomechanical properties of the sclera associated with a range of factors including age^4^,^5^,^6^, refractive error^2^,^3^,^7^,^8^ and pathological conditions such as glaucoma^6^,^7^. In the anterior eye, the sclera is covered by the conjunctiva, a translucent mucous membrane consisting of an epithelial lining and stromal layers of loose connective and lymphoid tissue^9^,^10^,^11^. The conjunctiva plays important roles in the eye’s mechanical and immune defence^12^ and in producing tear film components^10^.

Although *in vivo* measurements of the sclera and conjunctiva are possible with magnetic resonance imaging (MRI)^7^, and ultrasound biomicroscopy (UBM)^13^, these techniques either have relatively low resolution (MRI) or require contact with the eye (UBM). Anterior segment optical coherence tomography (AS-OCT) however, allows the *in vivo* anterior sclera^14^ and overlying conjunctiva^15^ to be imaged non-invasively and with high resolution. A number of recent studies have used AS-OCT imaging technology to document the changes in the anterior scleral structure associated with the presence of glaucoma^16^, following ocular surgery^17^ and therapeutic procedures^18^,^19^, contact lens wear^20^,^21^ and the presence of inflammatory conditions such as scleritis and episcleritis^22^.

The *in vivo* thickness of the anterior sclera of normal healthy adult eyes has also been documented by a small number of recent studies using AS-OCT^14^,^23^,^24^,^25^. These studies have typically assessed scleral thickness at a small number of discrete locations, in a range of different meridians of the anterior eye. Significant variations in the thickness of the anterior sclera have been reported to be associated with measurement location^14^,^23^, age^14^ and gender^23^,^25^. Although there are reports of reduced thickness of the posterior sclera associated with myopia^7^,^8^, studies of adults with a range of refractive errors have not found evidence of significant *in vivo* anterior scleral thinning associated with myopia^14^,^23^,^24^. To date, studies of the anterior sclera have focussed exclusively on measurements of adult subjects (with ages of participants ranging from 18 to 92 years), with no reports of anterior scleral thickness in paediatric populations.

A small number of recent studies have also documented the thickness of the normal anterior conjunctiva^15^,^26^,^27^,^28^,^29^. The thickness of the bulbar conjunctival epithelium has been shown to vary according to measurement meridian in a small population of normal young adults (being thickest inferiorly), but not in middle aged adults^27^. Zhang *et al*.^28^ examined the bulbar conjunctival thickness (including the conjunctival epithelium and stroma) in a large population of Chinese subjects (ranging in age from 8–85 years), and found that significant decreases in conjunctival thickness occurred with increasing age, however this study was limited to measurements from a single infero-temporal location approximately 3 mm from the limbus. Howlett *et al*.^29^ also found a decrease in the superior bulbar conjunctival thickness with age in a smaller population of mainly older adults in the UK.

Improved knowledge of the normal *in vivo* dimensions of the anterior sclera and conjunctiva is important for a range of applications such as the planning and management of ocular surgical and therapeutic procedures^17^,^18^,^19^, ocular drug delivery^30^, contact lens fitting and management^31^, as well as in the diagnosis of scleral and conjunctival pathology^2^. Advancing understanding of the normal *in vivo* anterior scleral dimensions will further our basic understanding of the anatomical characteristics of the anterior eye, and may also provide insights into the potential role of the sclera in human refractive error^2^ and in pathological conditions such as glaucoma^7^. Given the limited number of studies examining the anterior sclera and conjunctiva *in vivo*, and the paucity of data describing the anterior sclera in paediatric populations, in this study we have examined the normal thickness profile of the anterior sclera and conjunctiva in a population of healthy children and young adults with a range of refractive errors. We have documented the regional variations occurring in anterior tissue thickness, and explored the changes in thickness associated with refractive error, gender and age in this population.

## Methods

In this study, the thickness of the anterior sclera and conjunctiva was assessed using spectral domain AS-OCT in 111 subjects aged between 10 and 31 years of age (mean ± SD age 18.0 ± 5.4 years). All participants exhibited normal best corrected visual acuity in each eye, and had no history or evidence of ocular disease, injury or surgery. Fifty four percent of the participants were female. All participants in the study were of Caucasian ethnic origin. None of the participants were rigid contact lens wearers, and any soft contact lens wearers (n = 17) abstained from lens wear for 1 week prior to their involvement in the study to limit the influence of contact lens related scleral changes^20^ upon the results. Approval from the Queensland University of Technology human research ethics committee was provided prior to commencement of the study, and all experimental methods were carried out in accordance with the approved guidelines. Written informed consent was obtained from all adult participants, and written parental consent and child assent from all paediatric participants.

Prior to participation, all subjects underwent a screening examination to determine their visual and refractive status, and to assess their ocular health. Each participant’s sphero-cylindrical refractive error was determined using a non-cycloplegic subjective refraction, aiming for a maximum plus/minimum minus for best visual acuity endpoint. Ocular axial biometry was also measured for each participant using the Lenstar LS900 optical biometer (Haag Streit AG, Koeniz, Switzerland). The participants exhibited a range of refractive errors, with the mean spherical equivalent refraction (SER) of the right eye for all 111 subjects being −1.10 ± 1.90 D (range: +1.25 to −8.00 D), and the mean cylindrical refraction being −0.22 ± 0.34 D (range: 0.00 to −1.50 D). Each subjects’ right eye SER was used to categorise them as either a myope (SER ≤ −0.75 D, n = 46) or a non-myope (SER −0.375 to + 1.25, n = 65). Participants were also categorised according to their age as children (<18 years, n = 56) or adults (>18 years, n = 55). The demographic and ocular characteristics of the participants is summarised in Table 1.

The thickness of the temporal and nasal anterior conjunctiva and sclera of the right eye of each participant was derived from OCT images captured using the Heidelberg Spectralis OCT (Heidelberg Engineering, Heidelberg, Germany) device with anterior segment imaging module. This device captures cross-sectional images of the anterior eye with a scanning speed of 40,000 A scans/second, axial resolution of 3.9 μm and transverse resolution of 14 μm, using a super luminescent diode of central wavelength 870 nm. Each subject had high resolution images (each cross-sectional image consisting of 1536 by 678 pixels) of the anterior eye captured using the instrument’s scleral imaging mode with enhanced depth imaging to optimise the image of the sclera. For each subject, two scans of the temporal and two of the nasal anterior sclera and conjunctiva were captured, using a single 30° (16 mm) long line scan. All images were captured using the instrument’s automatic real time eye tracking (ART), to facilitate frame averaging to improve image signal to noise ratio, and each OCT cross-sectional image was the average of 50 B-Scans. During imaging, subjects fixated upon an external fixation target (a green LED placed at a distance of 4 metres from the subject’s eye and placed off-axis by 30° nasally or temporally). In order to promote a consistent scanning location within and between subjects, care was taken to include the peripheral cornea in all images and to align the line scan close to the lower edge of the bright corneal reflection in the en-face image. Although vertical alignment of images to the centre of the bright reflection in the OCT image would achieve a more precise imaging location, the lower edge of the reflection in the en-face image was employed for alignment in our current study to avoid the obscuration of image detail by large reflections in the scleral region that are often observed in images aligned to the centre of the specular reflection in the B-scan. Only scans with image quality index (QI) of >25 dB were included for analysis (mean image quality was 42.0 ± 3.6 dB). All measurements in the study were conducted between 9 am and 5 pm.

### Data Analysis

**Figure 1:**
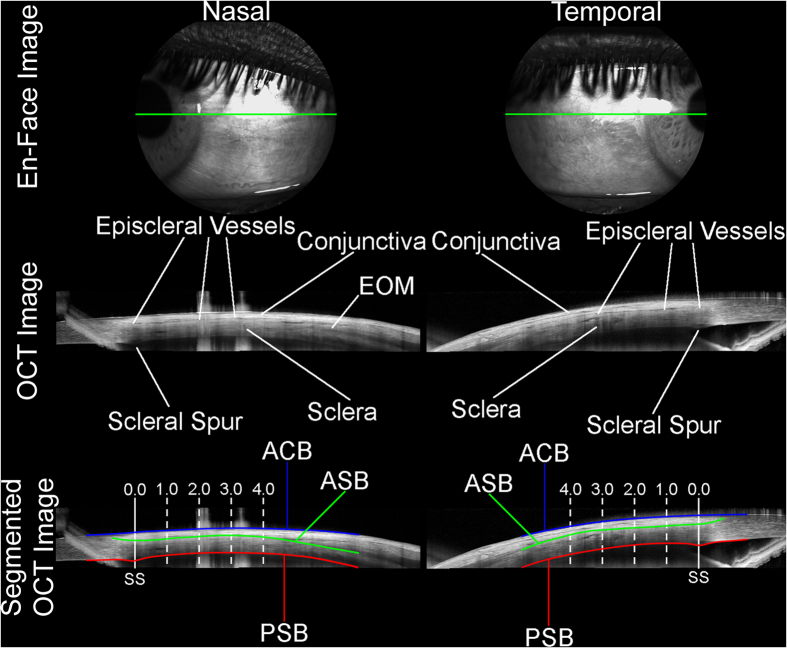
Illustration of the anterior eye OCT imaging performed on each subject. Images of the anterior sclera and conjunctiva were collected for each subject in the limbal region of the nasal (left) and temporal (right) meridians. Following image capture, the OCT images were analysed using a semi-automated procedure to delineate the anterior conjunctival boundary (blue line, ACB), the anterior scleral boundary (green line, ASB) and the posterior scleral boundary (red line, PSB). The location of the scleral spur (SS) was also marked manually. After correction for refractive distortion at each tissue boundary, scleral thickness (ASB to PSB) and conjunctival thickness (ACB to ASB) were calculated based upon the segmentation at 0.0 mm, 1.0 mm, 2.0 mm, 3.0 mm and 4.0 mm (measured along the arc of the curved posterior scleral boundary) from the SS, assuming a tissue refractive index of 1.41 for the sclera and 1.38 for the conjunctiva. In cases where the extraocular muscles (EOM) were visible (e.g. nasal OCT image), the ASB was placed on the outer portion of the sclera, effectively excluding the EOM from the scleral thickness and including the EOM as part of the conjunctival thickness measurement.

Following image acquisition, the OCT images of each participant were exported and analysed with a semi-automated procedure using custom written software. Figure 1 provides an overview of the OCT imaging and analysis procedures used in the study. Initially, an automated, graph based^32^,^33^ segmentation algorithm was used to delineate the anterior conjunctival boundary (ACB) and posterior scleral boundary (PSB). An independent observer, masked to the subjects’ demographic and ocular details, then analysed all images to correct any errors in the automated segmentation, and to manually segment the anterior scleral boundary (ASB, demarcated by the posterior edge of the layer of hypo-reflective episcleral blood vessels). The observer also manually marked the location of the scleral spur (the anterior inner termination point of the scleral tissue) in each image using the ciliary muscle method that has been previously described^34^.

The exported anterior segment OCT images are scaled by the Spectralis device to allow tissue thickness measures, assuming a uniform tissue refractive index of 1.40, and are not corrected for refractive distortion occurring at each of the tissue boundaries. In order to allow more reliable between subject tissue thickness comparisons, additional analysis was performed on each subject’s OCT image segmentation data, to correct the boundary locations for the effects of refraction at each of the tissue boundaries (using an approach based on Snell’s law, similar to that presented by Tian *et al*.^35^) and assuming a refractive index of 1.38 for conjunctival tissue and 1.41 for scleral tissue^36^. These refractive distortion corrected data, were then used to calculate the conjunctival thickness (the axial/vertical distance from the ACB to the ASB) and scleral thickness (the axial/vertical distance from the ASB to the PSB), across the length of each scan. The conjunctival thickness measurement therefore encompasses the conjunctival epithelium, conjunctival stroma and the episclera. Data from the two repeated scans in each meridian were averaged, and the temporal and nasal conjunctival and scleral thickness profiles were derived for each subject, using the scleral spur as the origin. For the purposes of statistical analysis, the conjunctival and scleral thickness at locations 0 mm, 1 mm, 2 mm, 3 mm and 4 mm (measured along the arc of the curved posterior scleral boundary) from the scleral spur in the temporal and nasal meridians for each subject were extracted from each thickness profile.

### Statistical Analysis

The scleral and conjunctival thickness data were not found to depart significantly from a normal distribution (Kolmogorov-Smirnov test, all p > 0.1 for the scleral and conjunctival thickness at all locations), and therefore parametric statistics were used throughout. To examine the scleral and conjunctival thickness data in this population, a repeated measures ANOVA was used, with two within-subject factors: measurement meridian (i.e. temporal or nasal), and measurement location with respect to the scleral spur (0 mm, 1 mm, 2 mm, 3 mm or 4 mm) and three between-subject factors: age group (children or adults), refractive group (myope or non-myope) and gender. Bonferroni corrected pairwise comparisons were carried out for any significant main effects and interactions. Additional repeated measures ANOVAs were also carried out including the continuous variables of age and spherical equivalent refractive error as covariates. We also used a stepwise multiple linear regression analysis to examine the association between demographic (age, gender) and biometric factors (axial length, anterior chamber depth, lens thickness and central corneal thickness) and the conjunctival and scleral thickness measures (the average thickness across all locations and meridians). P-values of <0.05 were considered statistically significant.

The repeatability and reliability of the thickness measures were assessed through analysis of the two repeated measures collected for each subject. The intraclass correlation coefficient (ICC) and the mean difference (and standard deviation) between the two repeated thickness measures was determined. This analysis was carried out for each of the measurement locations and meridians for scleral and conjunctival thickness, and revealed a high level of reliability for both the nasal and temporal thickness measures across all locations. Repeatability was further assessed by repeating the imaging and measurement procedure 10 times on one subject, and the ICC and within-subject standard deviation of the repeated thickness measures were calculated. This analysis revealed the conjunctival thickness measures to have an ICC of 0.934 and a within-subject standard deviation of 9.4 μm, and the scleral thickness measures to have an ICC of 0.977 and a within-subject standard deviation of 10.7 μm, indicative of a high level of repeatability for both the conjunctiva and sclera.

For the two repeated measures collected on each subject, the ICC for the conjunctival thickness data was >0.95 for all measurement locations over the two measured meridians (range: 0.954 to 0.989). Similarly, for the scleral thickness, the ICC was also >0.95 (range: 0.951 to 0.988). The mean thickness difference between repeated measures (and the standard deviation of these differences) was also small for all measurement locations for each meridian, indicative of good within-session repeatability for the scleral and conjunctival thickness measures. For conjunctival thickness, the mean thickness difference between repeated measures was less than ±3.2 μm (range: −2.5 to +3.1 μm) for all measurement locations in each meridian, with the standard deviation of the differences ranging from 11.6 to 25.6 μm. The mean difference between repeated measures for scleral thickness was less than ±3.0 μm (range: −2.2 to +3.0 μm), with standard deviation ranging from 11.6 to 25.0 μm.

## Results

### Average conjunctival and scleral thickness in children and young adults

**Figure 2:**
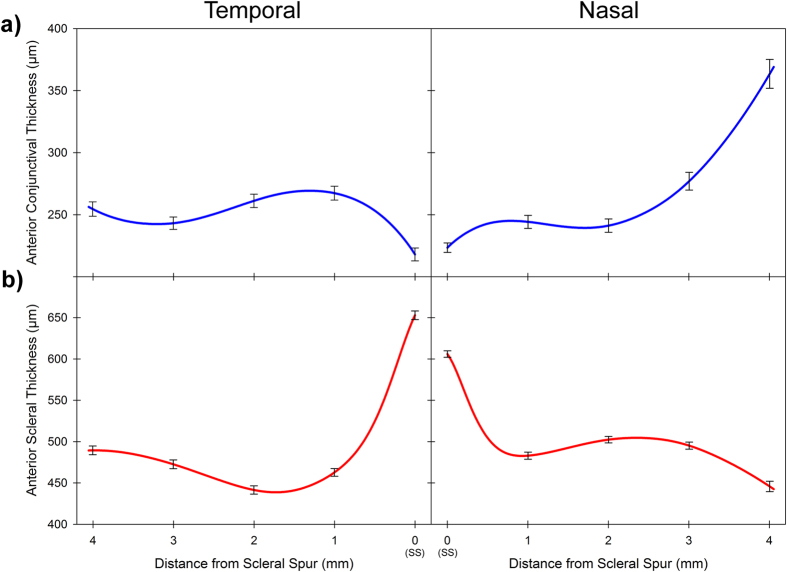
Average thickness profile of the temporal (left) and nasal (right) meridian with distance from the scleral spur (SS), for the anterior conjunctiva (blue line) (**a**) and the anterior sclera (red line) (**b**) for all 111 subjects in the study. Error bars represent the standard error of the mean, and are shown for each 1 mm distance from the scleral spur.

The average thickness profile of the anterior conjunctiva and sclera, in this population of 111 children and young adults is illustrated in Fig. 2 and Table 2. Repeated measures ANOVA revealed that the conjunctival thickness varied significantly with both measurement meridian and location, and also showed a significant meridian by location interaction (all p < 0.001). On average the conjunctiva was significantly thicker in the nasal meridian (mean thickness across all locations 270 ± 90 μm) compared to the temporal meridian (mean thickness 249 ± 59 μm) (p < 0.001). The conjunctiva exhibited its minimum thickness at the scleral spur location (0 mm) for both the temporal (mean 218 ± 55 μm) and nasal meridians (mean 223 ± 40 μm) however the pattern of change in thickness away from the scleral spur differed between the two meridians. For the temporal meridian, the conjunctiva increased to its maximum thickness at the 1 mm location (mean 267 ± 59 μm), whereas the nasal meridian exhibited its maximum thickness (mean 364 ± 122 μm) at the 4 mm location (Fig. 2, top).

Anterior scleral thickness also showed significant variations with measurement location, and a location by meridian interaction (both p < 0.001), but the average thickness of the nasal (mean thickness across all locations 506 ± 72 μm) and temporal meridians (504 ± 93 μm) were not significantly different. The sclera was thickest at the scleral spur location in both the temporal (653 ± 54 μm) and nasal (606 ± 42 μm) meridians and was significantly thinner at all locations away from the scleral spur (all comparisons p < 0.05). The nasal meridian was found to exhibit its minimum in thickness at the 4 mm location (446 ± 66 μm), whereas the temporal meridian exhibited a minimum in scleral thickness at the 2 mm location (442 ± 52 μm) (Fig. 2, bottom).

**Figure 3:**
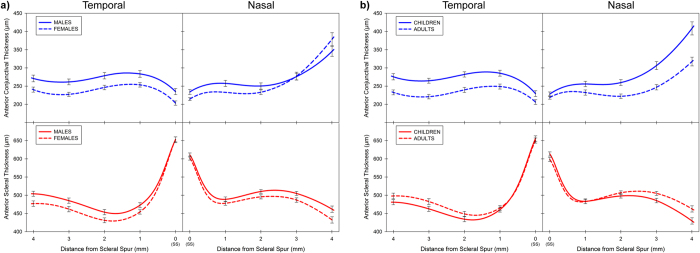
Average thickness profile of the temporal (left) and nasal (right) meridian with distance from the scleral spur, of the anterior conjunctiva (blue lines, top) and the anterior sclera (red lines, bottom) according to gender (**a**) and age (**b**). Thickness profiles for males (n = 51, solid lines) and females (n = 60, dashed lines) (**a**) and for children (n = 56, solid lines) and adults (n = 55, dashed lines) (**b**) are illustrated. Error bars represent the standard error of the mean, and are shown for each 1 mm distance from the scleral spur.

Significant differences in scleral thickness were also found to be associated with gender, with males (mean thickness 514 ± 34 μm) exhibiting significantly thicker sclera’s compared to females (mean 497 ± 35 μm) (p = 0.004), and the magnitude of the differences between males and females increased with increasing distance from the scleral spur (gender by location interaction p = 0.03) (Fig. 3a). The conjunctival thickness was also on average thicker in males (mean 269 ± 49 μm) compared to females (mean 251 ± 43 μm), although this difference didn’t reach statistical significance (p = 0.24). However, significantly greater conjunctival thickness in males was found in the temporal meridian (meridian by gender interaction p = 0.01) and in locations (0 mm, 1 mm and 2 mm) closer to the scleral spur (location by gender interaction (p = 0.02).

Age was also a significant factor influencing scleral and conjunctival thickness (Fig. 3b). Children were found to have significantly thicker anterior conjunctivas (mean thickness across all locations 280 ± 45 μm) compared to adults (mean 239 ± 38 μm) (p < 0.001), and the magnitude of these differences increased with greater distance away from the scleral spur (age group by location interaction, p < 0.001). In contrast to the conjunctival thickness findings, children exhibited on average a thinner sclera (mean thickness across all locations 500 ± 36 μm) than the adults (mean 510 ± 34 μm), although this difference did not reach statistical significance (p = 0.114). However, there was a significant age group by measurement location interaction (p = 0.001) for the sclera, with the children exhibiting significantly thinner anterior sclera’s compared to adults at both the 3 mm and 4 mm locations (each p < 0.01), but not at the other locations (all p > 0.05). Analyses including age as a continuous covariate gave similar outcomes, with significant age, and age by measurement location (each p < 0.05) interactions observed for both conjunctival and scleral thickness, with younger age being associated with a thicker conjunctiva and a thinner sclera. There was no significant age by gender interaction for either conjunctival thickness or scleral thickness (all p > 0.05).

Neither analyses including refractive group as a between subject factor or analyses including refractive error as a continuous covariate revealed a significant effect of refractive error upon the anterior scleral or conjunctival thickness (all p > 0.05). There were also no significant interactions between refractive group and measurement meridian and/or measurement location, and no interactions between refractive group and age and/or gender (all p > 0.05).

**Figure 4:**
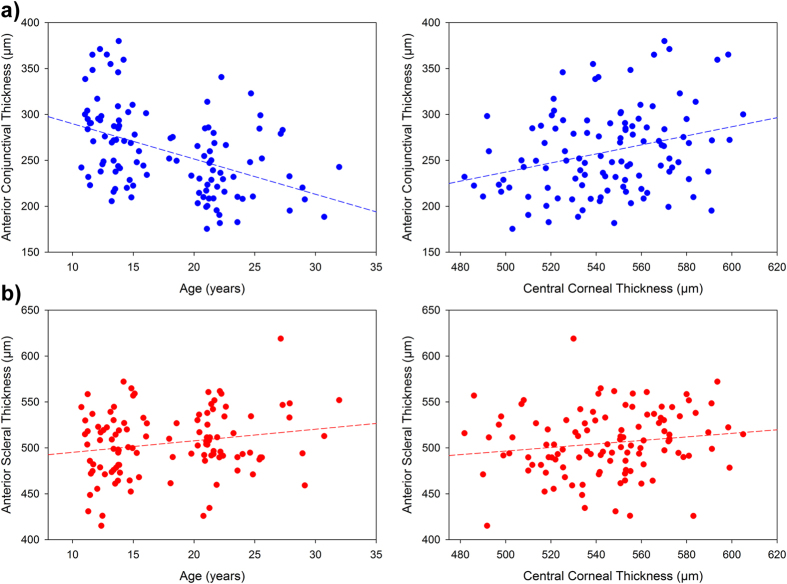
Scatterplots illustrating the relationship between thickness and both age (left) and CCT (right). Data for both global anterior conjunctival thickness (averaged across all locations and meridians) (blue symbols, **a**) and global anterior scleral thickness (red symbols, **b**) are shown. Dashed line indicates best fit linear regression line.

Stepwise multiple regression analysis (Table 3, Fig. 4) revealed that the mean anterior conjunctival thickness was significantly negatively associated with age (β = −3.4, p < 0.001) and positively associated with central corneal thickness (CCT) (β = 0.308, p = 0.04). The mean anterior scleral thickness was significantly positively associated with both age (β = 2.4, p < 0.001) and CCT (β = 0.413, p = 0.001) and anterior chamber depth (ACD) (β = 0.046, p < 0.001). Neither axial length, nor spherical equivalent refractive error were significantly associated with either anterior conjunctival thickness or anterior scleral thickness (each p > 0.05).

## Discussion

This study reports upon the average *in vivo* thickness profile of the anterior sclera and conjunctiva in children and young adults using spectral domain AS-OCT, and demonstrates that significant variations in the thickness of both the anterior sclera and conjunctiva occur with age, gender and measurement location. Although a number of recent studies have investigated scleral thickness in adults, to our knowledge this study provides the first report of the anterior scleral thickness in paediatric subjects, and contributes to our understanding of the normal dimensions of the anterior sclera and conjunctiva and the factors that can influence these dimensions in young populations.

Both scleral and conjunctival thickness exhibited significant variations dependent upon measurement location. The pattern of thickness change that we observed in the anterior sclera with increasing distance from the scleral spur, is generally consistent with previous reports of both *in vivo*^13^,^23^,^24^ and *ex vivo*^7^,^8^ scleral thickness using a variety of measurement techniques, where the sclera in the anterior segment has typically been documented to show a maximum near the scleral spur and then a reduction posterior to this location. Previous studies of total bulbar conjunctival thickness have only assessed thickness at a single discrete location approximately 3 mm inferotemporal^15^,^28^ or superior^29^ to the limbus. However, the variations in conjunctival thickness that we observed, with the conjunctiva being thinnest at the scleral spur location, are consistent with the conjunctiva’s anatomical characteristics, with the bulbar conjunctiva terminating at the corneo-scleral junction. The significant variations observed in conjunctival thickness with distance from the scleral spur, highlights the importance of knowledge of measurement location when assessing anterior eye tissue thickness.

The nasal-temporal asymmetries observed in scleral and conjunctival thickness, with the sclera being thinner, and conjunctiva thicker in the nasal meridian compared to the temporal meridian, particularly in the locations 4 mm from the scleral spur, are consistent with some of the previously documented anatomical features of the anterior segment^37^,^38^,^39^. These meridional differences in thickness appear to be at least partly explained by the locations of the medial and lateral rectus extraocular muscles (EOMs) that insert into the sclera near the equatorial region of the eye globe. It is well documented that the medial rectus inserts at a location more proximal to the limbus compared to the lateral rectus insertion^37^,^38^,^39^. In a study of 30 normal adults, Park *et al*.^39^ reported the mean distance from the limbus to the lateral rectus insertion to be 6.0 ± 0.6 mm (range: 4.8 to 7.9 mm), and the limbus to the medial rectus insertion to be 5.4 ± 1.4 mm (range: 3.1 to 7.0 mm), which suggests that the presence of the extraocular muscles (EOM) is likely to impact the more distal thickness measurements, particularly on the nasal side (e.g. Fig. 1). With our segmentation approach for delineating the ASB, the presence of the EOM would contribute to an increase in conjunctival thickness. A more proximal insertion of the medial rectus compared to the lateral rectus would therefore be expected to result in the nasal scleral measurement being thinner and nasal conjunctiva measurement being thicker compared to the temporal region, as was observed.

Significant changes in scleral thickness were also found with age, with an increase in scleral thickness observed from childhood into early adulthood, which was more prominently observed in locations distal from the scleral spur. Although the exact mechanism underlying these age-related changes in the anterior sclera is not clear, our results are consistent with previous *in-vitro* work that has shown an increase in scleral proteoglycans occurs from infancy up to the fourth decade of life, indicating that the sclera is a dynamic structure during normal ocular growth^4^. Recently, using AS-OCT, Ebneter *et al*.^14^ also reported a significant increase in the *in vivo* anterior scleral thickness with age in Caucasian adults (aged from 18 to 92 years). It should be noted that the changes associated with age observed in our current study were of small magnitude (estimated increase in anterior scleral thickness of ~2 μm per year), and the general pattern of thickness variation with distance from the scleral spur observed in children paralleled the pattern observed in the adults. This suggests that the dimensions of the sclera are closely approaching adult levels in the paediatric age group we have examined.

The conjunctival thickness measures also showed some significant changes with age, but in contrast to the sclera, the conjunctiva was found to reduce in thickness from childhood into early adulthood. A similar finding was also reported by Zhang *et al*.^28^, with reductions in conjunctival thickness observed in their *in vivo* conjunctival thickness measures in Chinese subjects aged from 8–92 years. These age-related decreases in conjunctival thickness are consistent with some previously documented features of conjunctival tissue from histological studies, with conjunctival goblet cell density showing a peak in infancy^10^ and conjunctival lymphoid tissue^9^ showing a peak in childhood and then both reducing into early adulthood.

Interestingly, we also observed significant effects of gender upon the thickness data, with greater scleral and conjunctival thickness observed in male subjects. Although not a universal finding in all studies^14^, a thicker anterior sclera in males has been reported by a number of previous *in vivo* studies using a variety of measurement techniques^23^,^25^,^40^. The small number of studies of *in vivo* conjunctival thickness to date^15^,^28^,^29^, have not found significant differences in conjunctival thickness associated with gender. However, it should be noted that these previous studies measured the conjunctiva at a single location approximately 3 mm from the limbus, and our current study did not find significant gender differences at all locations, with significant differences more prominent at regions 2 mm and closer to the scleral spur. Our findings suggest that hormonal differences may impact on conjunctival thickness in younger populations. Although our subjects did not exhibit evidence or history of conjunctival abnormalities, it is interesting to note that there is also evidence for hormonal influences in certain conjunctival conditions such as allergic conjunctivitis and related conditions^41^,^42^ which are reported to be substantially more prevalent in young male patients.

In our current study of children and young adults, we found no significant association between anterior scleral thickness and the presence and/or the magnitude of myopia, which is consistent with a number of previous studies of the *in vivo* anterior sclera in adult populations^16^,^23^,^24^. Likewise, axial length (the major biometric correlate of refractive error) was also not significantly associated with anterior scleral thickness in our population. These findings support the notion that any scleral thinning associated with myopia is primarily confined to more posterior regions of the globe^7^,^8^.

Although this study provides the first report of anterior scleral thickness measures in children, a limitation of this work is the relatively narrow range of ages examined, and the fact that only nasal and temporal meridians were imaged. It will therefore be of interest in future work to examine larger populations of subjects with a wider spectrum of ages (including younger children and older adults) across a wider range of scleral meridians and locations in order to more comprehensively characterise the developmental and aging changes occurring in the anterior sclera. An additional limitation of this study, is the fact that measurements were collected between 9 am and 5 pm, which leaves the possibility that diurnal changes in the sclera and/or conjunctiva may have impacted upon the results. However, in our recent study of diurnal variations in anterior scleral and conjunctival thickness^43^, we found the largest diurnal changes occurred in the early morning immediately after waking, with only small magnitude changes (that were typically not statistically significant) evident between 9 am and 5 pm. Furthermore, additional analyses including imaging time of day as a covariate did not reveal a significant association between time of day and scleral or conjunctival thickness in our current study (both p > 0.05). This suggests that diurnal variations are unlikely to have been a significant confounder.

In our current study, an axial thickness metric was used to define the scleral and conjunctival thickness (i.e. thickness measures were taken along the vertical direction of the image between the reference boundaries of interest), since axial definitions of thickness are most commonly used in ophthalmic OCT^44^. However, it should be noted that these thickness metrics could be influenced by rotation/tilt of the image. Alternative thickness metrics, such as thickness along a normal to one of the boundaries would be less likely to be influenced by image tilt or rotation (but could potentially be more affected by local curvature changes along the boundary of interest as occurs commonly at the scleral spur for instance). To explore the reliability of the axial thickness metrics used, we re-calculated all of the scleral and conjunctival thickness measures for all subjects along a normal to the scleral boundary. The average difference between the ‘axial’ thickness and the ‘normal’ thickness measures was small (mean difference of 0.6 ± 6.3 μm and 1.3 ± 3.3 μm for scleral and conjunctival thickness respectively), suggesting that the axial thickness metric was not substantially biased by image tilt/rotation in our analysis. Our imaging approach of aligning each OCT scan vertically with the lower edge of the corneal reflection in the en-face view may also have resulted in some variability in the vertical positioning of the B-scan images. However, the high repeatability found with our imaging and measurement procedure suggests that any variability in scan position was small.

An advantage of our analysis approach in this study is that correction for refractive distortion at the tissue boundaries was applied to the data, individual refractive indices were used for the scleral and conjunctival tissues, and measurement locations were defined based upon measures along the arc of the curved posterior sclera. This analysis approach should provide more robust between subject comparisons (particularly where subjects exhibit differences in eye size and curvature of the anterior segment) compared to analyses not correcting for refractive distortion and using straight line transverse distances to define measurement locations. To examine the influence of our analysis approach on thickness measures, we re-analysed the data without correcting for refractive distortion, assuming a single tissue refractive index (1.40) and with straight line distances defining the measurement locations. For the temporal images, the mean difference between our final distortion corrected analysis and the uncorrected analysis was −6.8 ± 1.2 μm for the scleral and 3.2 ± 1.2 μm for the conjunctival thickness (across all measurement locations). The mean difference for the nasal images was −6.6 ± 1.7 μm and 2.6 ± 1.9 μm for the sclera and conjunctiva respectively. The small magnitude of these differences suggests only minor effects of refractive distortion upon the thickness findings, where not accounting for these issues results on average in −1.3% and +1.1% error in the scleral and conjunctival thickness estimates respectively.

In conclusion, this study reports the average thickness profile of the anterior sclera and conjunctiva in normal children and young adults, and demonstrates that significant variations in scleral and conjunctival thickness occur with age, gender and with measurement location. Refractive error however, does not appear to significantly influence the anterior scleral (or conjunctival) thickness in this population of young subjects.

## Additional Information

**How to cite this article**: Read, S. A. *et al*. Anterior eye tissue morphology: Scleral and conjunctival thickness in children and young adults. *Sci. Rep.* **6**, 33796; doi: 10.1038/srep33796 (2016).

## Figures and Tables

**Table 1:** Mean ± SD demographic and ocular characteristics of the study participants, categorised according to refractive group and age.

Category		Age (years)	SER (D)	% Female	n
Refractive Group	Myopes	18.6 ± 5.1	−2.90 ± 1.74	63%	46
	Non-Myopes	17.5 ± 5.5	0.17 ± 0.34	48%	65
Age Group	Children	13.2 ± 1.4	−0.64 ± 1.61	46%	56
	Adults	22.8 ± 3.1	−1.56 ± 2.07	62%	55
All Subjects		18.0 ± 5.4	−1.10 ± 1.90	54%	111

SER denotes spherical equivalent refraction.

**Table 2:** Mean thickness of the anterior conjunctiva and anterior sclera with distance from the scleral spur (SS) in the temporal and nasal meridians for all subjects in the study (n = 111).

		Mean ± SD thickness (μm)				
		0 mm (SS)	1 mm	2 mm	3 mm	4 mm
Anterior Conjunctiva	Temporal	218 ± 55	267 ± 59	261 ± 57	243 ± 52	255 ± 61
	Nasal	223 ± 40	244 ± 56	241 ± 57	277 ± 75	364 ± 122
Anterior Sclera	Temporal	653 ± 54	463 ± 49	442 ± 52	473 ± 56	489 ± 56
	Nasal	606 ± 42	483 ± 45	502 ± 41	495 ± 45	446 ± 66

**Table 3:** Overview of results from stepwise multiple linear regression analysis examining the significant demographic and biometric associations with the average anterior conjunctival and scleral thickness.

		Unstandardized Coefficient ‘B’ (Standard Error)	Significance	Model R^2^ (P-Value)
Anterior Conjunctival Thickness	Age	−3.4 (0.8)	<0.001	0.228 (<0.001)
	CCT	0.308 (0.149)	0.04	
Anterior Scleral Thickness	Gender	−16.6 (6.0)	0.007	0.254 (<0.001)
	Age	2.4 (0.6)	<0.001	
	CCT	0.413 (0.128)	0.001	
	ACD	0.046 (0.013)	<0.001	

CCT (central corneal thickness), ACD (anterior chamber depth).

## References

[b1] Watson P. G. & Young R. D. Scleral structure, organisation and disease. Exp Eye Res. 78, 609–623 (2004). 15106941 10.1016/s0014-4835(03)00212-4

[b2] Summers Rada J. A., Shelton S. & Norton T. T. The sclera and myopia. Exp Eye Res. 82, 185–200 (2006). 16202407 10.1016/j.exer.2005.08.009

[b3] McBrien N. A., Jobling A. I. & Gentle A. Biomechanics of the sclera in myopia: Extracellular factors. Optom Vis Sci. 86, E23–E30 (2009). 19104466 10.1097/OPX.0b013e3181940669

[b4] Rada J. A., Achen V. R., Penugonda S., Schmidt R. W. & Mount B. A. Proteoglycan composition in the human sclera during growth and aging. Invest Ophthalmol Vis Sci. 41, 1639–1648 (2000). 10845580

[b5] Girard M. J. A., Suh J.-K. F., Bottlang M., Burgoyne C. F. & Crawford Downs J. Scleral biomechanics in the aging monkey eye. Invest Ophthalmol Vis Sci. 50, 5226–5237 (2009). 19494203 10.1167/iovs.08-3363 PMC2883469

[b6] Coudrillier B. . Biomechanics of the human posterior sclera: Age- and glaucoma-related changes measured using inflation testing. Invest Ophthalmol Vis Sci. 53, 1714–1728 (2012). 22395883 10.1167/iovs.11-8009 PMC3630906

[b7] Norman R. E. . Dimensions of the human sclera: Thickness measurement and regional changes with axial length. Exp Eye Res. 90, 277–284 (2010). 19900442 10.1016/j.exer.2009.11.001

[b8] Vurgese S., Panda-Jonas S. & Jonas J. B. Scleral thickness in human eyes. PLoS One. 7, e29692 (2012). 22238635 10.1371/journal.pone.0029692 PMC3253100

[b9] Osterlind G. An investigation into the presence of lymphatic tissue in the human conjunctiva, and its biological and clinical importance. Acta Ophthalmol. 23, 1–79 (1944).

[b10] Kessing S. V. Mucous gland system of the conjunctiva. A quantitative normal anatomical study. Acta Ophthalmol. 95, 1–133 (1968). 4170134

[b11] Efron N., Al-Dossari M. & Pritchard N. *In vivo* confocal microscopy of the bulbar conjunctiva. Clin Exp Ophthalmol. 37, 335–344 (2009). 19594558 10.1111/j.1442-9071.2009.02065.x

[b12] Knop N. & Knop E. Conjunctiva-associated lymphoid tissue in the human eye. Invest Ophthalmol Vis Sci. 41, 1270–1279 (2000). 10798640

[b13] Oliveira C., Tello C., Liebmann J. & Ritch R. Central corneal thickness is not related to anterior scleral thickness or axial length. J. Glaucoma. 15, 190–194 (2006). 16778639 10.1097/01.ijg.0000212220.42675.c5

[b14] Ebneter A., Haner N. U. & Zinkernagel M. S. Metrics of the normal anterior sclera: imaging with optical coherence tomography. Graefes Arch Clin Exp Ophthalmol. 253, 1578–1580 (2015). 10.1007/s00417-015-3072-5 PMC4548011 26067393

[b15] Zhang X. . *In vivo* cross-sectional observation and thickness measurement of bulbar conjunctiva using optical coherence tomography. Invest Ophthalmol Vis Sci. 52, 7787–7791 (2011). 21873655 10.1167/iovs.11-7749

[b16] Yoo C., Eom Y. S., Suh Y.-W. & Kim Y. Y. Central corneal thickness and anterior scleral thickness in Korean patients with open-angle glaucoma: an anterior segment optical coherence tomography study. J. Glaucoma. 20, 95–99 (2011). 20577104 10.1097/IJG.0b013e3181dde051

[b17] Leung C. K. S. . Analysis of bleb morphology after trabeculectomy with Visante anterior segment optical coherence tomography. Br J. Ophthalmol. 91, 340–344 (2007). 17005548 10.1136/bjo.2006.100321 PMC1857643

[b18] Taban M. . Scleral thickness following fluocinolone acetonide implant (Retisert). Ocul Immunol Inflamm. 18, 305–313 (2010). 20482407 10.3109/09273941003658292

[b19] Zinkernagel M. S., Schorno P., Ebneter A. & Wolf S. Scleral thinning after repeated intravitreal injections of endothelial growth factor agents in the same quadrant. Invest Ophthalmol Vis Sci. 56, 1894–1900 (2015). 25711641 10.1167/iovs.14-16204

[b20] Alonso-Caneiro D., Shaw A. J. & Collins M. J. Using optical coherence tomography to assess corneoscleral morphology after soft contact lens wear. Optom Vis Sci. 89, 1619–1622 (2012). 23034339 10.1097/OPX.0b013e31826c5f63

[b21] Alonso-Caneiro D., Vincent S. J. & Collins M. J. Morphological changes in the conjunctiva, episclera and sclera following short-term miniscleral contact lens wear in rigid lens neophytes. Contact Lens Ant Eye. 39, 53–61 (2016). 10.1016/j.clae.2015.06.008 26189941

[b22] Shoughy S. S., Jaroudi M. O., Kozak I. & Tabbara K. F. Optical coherence tomography in the diagnosis of scleritis and episcleritis. Am J. Ophthalmol. 159, 1045–1049 (2015). 25771347 10.1016/j.ajo.2015.03.004

[b23] Buckhurst H. D., Gilmartin B., Cubbidge R. P. & Logan N. S. Measurement of scleral thickness in humans using anterior segment optical coherence tomography. PLoS One. 10, e0132902 (2015). 26218188 10.1371/journal.pone.0132902 PMC4517791

[b24] Pekel G. . Comparison of corneal layers and anterior sclera in emmetropic and myopic eyes. Cornea. 34, 786–790 (2015). 25811725 10.1097/ICO.0000000000000422

[b25] Schlatter B., Beck M., Frueh B. E., Tappeiner C. & Zinkernagel M. Evaluation of scleral and corneal thickness in keratoconus patients. J. Cataract Refract Surg. 41, 1073–1080 (2015). 25935338 10.1016/j.jcrs.2014.08.035

[b26] Feng Y. & Simpson T. L. Corneal, limbal, and conjunctival epithelial thickness from optical coherence tomography. Optom Vis Sci. 85, E880–E883 (2008). 18772715 10.1097/OPX.0b013e318185272d

[b27] Francoz M., Karamoko I., Baudouin C. & Labbé A. Ocular surface epithelial thickness evaluation with spectral-domain optical coherence tomography. Invest Ophthalmol Vis Sci. 52, 9116–9123 (2011). 22025572 10.1167/iovs.11-7988

[b28] Zhang X. . Bulbar conjunctival thickness measurements with optical coherence tomography in healthy Chinese subjects. Invest Ophthalmol Vis Sci. 54, 4705–4709 (2013). 23744999 10.1167/iovs.12-11003

[b29] Howlett J., Vahdani K. & Rossiter J. Bulbar conjunctival and Tenon’s layer thickness measurement using optical coherence tomography. J. Curr Glaucoma Pract. 8, 63–66 (2014). 26997811 10.5005/jp-journals-10008-1163 PMC4741171

[b30] Lee S.-B., Geroski D. H., Prausnitz M. R. & Edelhauser H. F. Drug delivery through the sclera: effects of thickness, hydration and sustained release systems. Exp Eye Res. 78, 599–607 (2004). 15106940 10.1016/s0014-4835(03)00211-2

[b31] van der Worp E. . Modern scleral contact lenses: A review. Cont Lens Anterior Eye. 37, 240–250 (2014). 24631015 10.1016/j.clae.2014.02.002

[b32] Chiu S. J. . Automatic segmentation of seven retinal layers in SDOCT images congruent with expert manual segmentation. Opt. Express. 18, 19413–19428 (2010). 20940837 10.1364/OE.18.019413 PMC3408910

[b33] LaRocca F. . Robust automatic segmentation of corneal layer boundaries in SDOCT images using graph theory and dynamic programming. Biomed. Opt. Express. 2, 1524–1538 (2011). 21698016 10.1364/BOE.2.001524 PMC3114221

[b34] Seager F. E., Wang J., Arora K. S. & Quigley H. A. The effect of scleral spur identification methods on structural measurements by anterior segment optical coherence tomography. J. Glaucoma. 23, e29–e38 (2014). 23807354 10.1097/IJG.0b013e31829e55ae

[b35] Tian J., Marzialano P., Baskaran M., Wong H.-T. & Aung T. Automatic anterior chamber angle assessment for HD-OCT images. IEEE Trans Biomed Eng. 58, 3242–3249 (2011). 21880568 10.1109/TBME.2011.2166397

[b36] Nemati B., Rylander H. G. & Welch A. J. Optical properties of conjunctiva, sclera, and the ciliary body and their consequences for transscleral cyclophotocoagulation. Appl Opt. 35, 3321–3327 (1996). 21102718 10.1364/AO.35.003321

[b37] Swan K. C. & Wilkins J. H. Extraocular muscle surgery in early infancy- Anatomical factors. J. Pediatr Ophthalmol Strabismus. 21, 44–49 (1984). 6726554 10.3928/0191-3913-19840301-03

[b38] Liu X., Wang F., Xiao Y., Ye X. & Hou L. Measurement of the limbus-insertion distance in adult strabismus patients with anterior segment optical coherence tomography. Invest Ophthalmol Vis Sci. 52, 8370–8373 (2011). 21948556 10.1167/iovs.11-7752

[b39] Park K.-A., Lee J. Y. & Yeul S. Reproducibility of horizontal extraocular muscle insertion distance in anterior segment optical coherence tomography and the effect of head position. J. AAPOS. 18, 15–20 (2014). 24568976 10.1016/j.jaapos.2013.11.005

[b40] Mohamed-Noor J. . Correlation between corneal and scleral thickness in glaucoma. J. Glaucoma. 18, 32–36 (2009). 19142132 10.1097/IJG.0b013e31816b2fd1

[b41] Bonini S., Coassin M., Aronni S. & Lambiase A. Vernal keratoconjunctivitis. Eye. 18, 345–351 (2004). 15069427 10.1038/sj.eye.6700675

[b42] La Rosa M. . Allergic conjunctivitis: a comprehensive review of the literature. Ital J. Pediatr. 39, 18 (2013). 23497516 10.1186/1824-7288-39-18 PMC3640929

[b43] Read S. A. . Diurnal variation of anterior scleral and conjunctival thickness. Ophthalmic Physiol Opt. 36, 279–289 (2016). 26931410 10.1111/opo.12288

[b44] Alonso-Caneiro D., Read S. A., Vincent S. J., Collins M. J. & Wojtkowski M. Tissue thickness calculation in ocular optical coherence tomography. Biomed Opt Exp. 7, 629–645 (2016). 10.1364/BOE.7.000629 PMC4771476 26977367

